# Oxidative Folding of Proteins: The “Smoking Gun” of Glutathione

**DOI:** 10.3390/ijms221810148

**Published:** 2021-09-20

**Authors:** Alessio Bocedi, Giada Cattani, Giorgia Gambardella, Linda Schulte, Harald Schwalbe, Giorgio Ricci

**Affiliations:** 1 Department of Chemical Sciences and Technologies, University of Rome “Tor Vergata”, 00133 Rome, Italy; bcdlss01@uniroma2.it (A.B.); giada.cattani@gmail.com (G.C.); giorgia.gambardella@gmail.com (G.G.); 2 Center for Biomolecular Magnetic Resonance (BMRZ), Institute for Organic Chemistry and Chemical Biology, Goethe-University Frankfurt, 60438 Frankfurt, Germany; schulte@nmr.uni-frankfurt.de (L.S.); Schwalbe@nmr.uni-frankfurt.de (H.S.)

**Keywords:** oxidative folding, glutathionylation, nitrosylation, cysteine reactivity, ribosomal exit tunnel, transient complex, glutathione

## Abstract

Glutathione has long been suspected to be the primary low molecular weight compound present in all cells promoting the oxidative protein folding, but twenty years ago it was found “not guilty”. Now, new surprising evidence repeats its request to be the “smoking gun” which reopens the criminal trial revealing the crucial involvement of this tripeptide.

## 1. Introduction

For many years the oxidized form of glutathione (GSSG) was considered the main culprit for the oxidative folding of many proteins. Indeed, GSSG displays an unusually high concentration in the endoplasmic reticulum. Further, its role in establishing the cellular redox potential is undisputed. In addition, a few disulfide containing proteins, when reduced and incubated with a GSH/GSSG mixture in a ratio similar to the one found in this cell compartment, refolded, correctly forming native disulfides. However, twenty years ago Cuozzo and Kaiser [1] claimed that GSSG cannot be considered the culprit because, when the cell is deprived of this compound, oxidative folding still occurs. At this stage, ER oxidoreductin 1 (Ero1) and the protein disulfide isomerase (PDI) were indicated as the main responsible for protein folding [1]. This hypothesis was rapidly accepted by the scientific community although conflicting evidence emerged from Kaiser’s own study. In fact, why does the disulfide bond formation still occur in cells that are simultaneously defective in both glutathione biosynthesis and Ero1 function? Bardwell and co-workers, in an interesting comment on these results, postulated the existence of a second, yet-to-be discovered oxidizing pathway [2]. They concluded that the ultimate source of oxidizing equivalents for the protein disulfide formation still has to be identified and that it remains “a complete mystery” [2].

In this context, other comments were also instructive. By considering that the rate-limiting steps for native disulfide bond formation in vivo are the late, complex, isomerization steps, whereas oxidation is much more rapid [3], Freedman and co-workers concluded that “*there is no reason to exclude the possibility that GSSG is on the normal oxidative pathway for secretory proteins, since in the absence of GSSG a normally minor direct oxidative pathway may become the major pathway. In such a case, the overall rate of production of native proteins would not be compromised by the change in oxidation pathway as the oxidative steps are not themselves rate-limiting*” [4]. Despite these counterarguments, no striking evidence was able to reverse the Cuozzo and Kaiser dogma. As a consequence, in almost all recent reviews about oxidative folding, glutathione was only related as a redox regulating agent for PDI and no direct interaction of this compound with the nascent reduced protein was considered [5,6,7]. Now we can show surprising findings that could light up the crime scene, at least in its early phase, and that can reverse the previous sentence.

**Figure 1:**
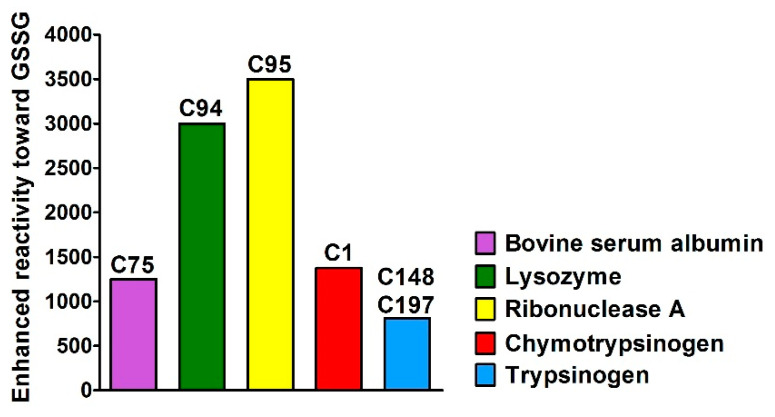
Hyper-reactivity of structural cysteines in five different proteins. Hyper-reactivity of Cys75, Cys94, Cys95, Cys1, Cys148 and Cys197 toward GSSG found in albumin, lysozyme, ribonuclease A, chymotrypsinogen, and trypsinogen, respectively. Pseudo first-order kinetic constants were normalized to that of an unperturbed protein cysteine.

Recently, we found that a few cysteines in the fully reduced albumin, adopting a molten globule-like conformation, showed unusual hyper-reactivity toward GSSG and various thiol reagents [8]. In particular, a single cysteine, identified as Cys75, displayed a second-order kinetic constant > 250 M^−1^ s^−1^ which corresponds to more than one thousand times higher reactivity toward GSSG than the one of an unperturbed protein cysteine (*k* = 0.2 M^−1^ s^−1^) (Figure 1) [9]. At first, we considered this surprising reactivity as a specific feature of a single protein. However, soon after this first observation, we discovered a similar, but even more striking hyper-reactivity in a cysteine (Cys94) of the reduced lysozyme in its unfolded state [10]. In this case, the reactivity toward GSSG was found to be more than 3000 times higher than that of a normal amino acid cysteine. We then hypothesized a possible function of this hyper-reactivity: when lysozyme lacks its four disulfides it rapidly collapses into irreversible and insoluble aggregates. The very fast reaction of Cys94 with GSSG inhibits instantaneously the aggregation [10]. This evidence gathered for a second protein represented a strong indication that this phenomenon was not a specific feature of albumin, as we initially thought, but could be a more general mechanism linked to protein folding.

Motivated by this observation, we searched for other hyper-reactive cysteines. We found a thousand times increased reactivity toward GSSG for Cys95, Cys1 and for both Cys148 and Cys197 in the reduced molten globule conformations of ribonuclease [11], chymotrypsinogen [12] and trypsinogen [13], respectively. In all these proteins the occurrence of a transient protein-GSSG complex was demonstrated on the basis of the quenching of intrinsic fluorescence occurring before the glutathionylation event in ribonuclease [11], lysozyme [10], chymotrypsinogen [12], and trypsinogen [13] (Table 1). The transient complex represents the origin of this unknown kinetic property.

**Figure 2:**
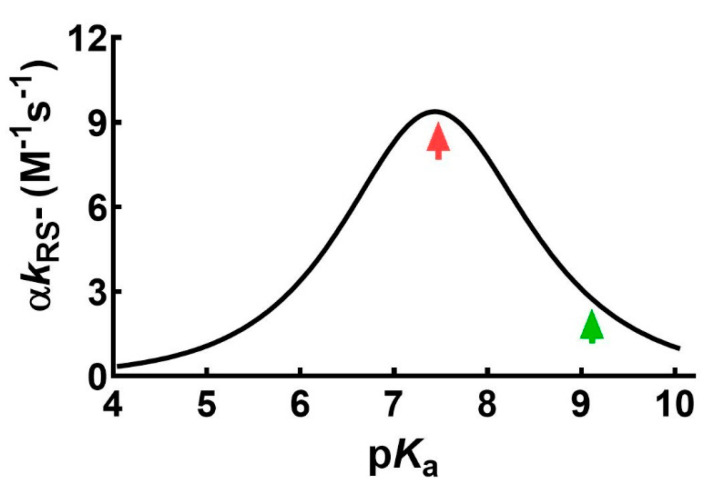
Dependence of the second-order kinetic constants (α*k*_RS_-) on p*K*_a_ for the reaction of several thiols with different p*K*_a_ with different disulfides at pH 7.4 (modified from Ref. [9]). The red arrow marks the maximum value of the bell-shaped graph. The p*K*_a_ of the unperturbed protein cysteine is labelled with the green arrow. The maximum implement of reaction rate due to a lowered p*K*_a_ was found to be 3 times.

A possible role in this phenomenon of a lowered p*K*_a_ of the sulfhydryl group was also considered but a recent investigation [9] likely demonstrated that a low p*K*_a_ cannot produce more than three times increased reactivity toward GSSG (Figure 2).

**Figure 3:**
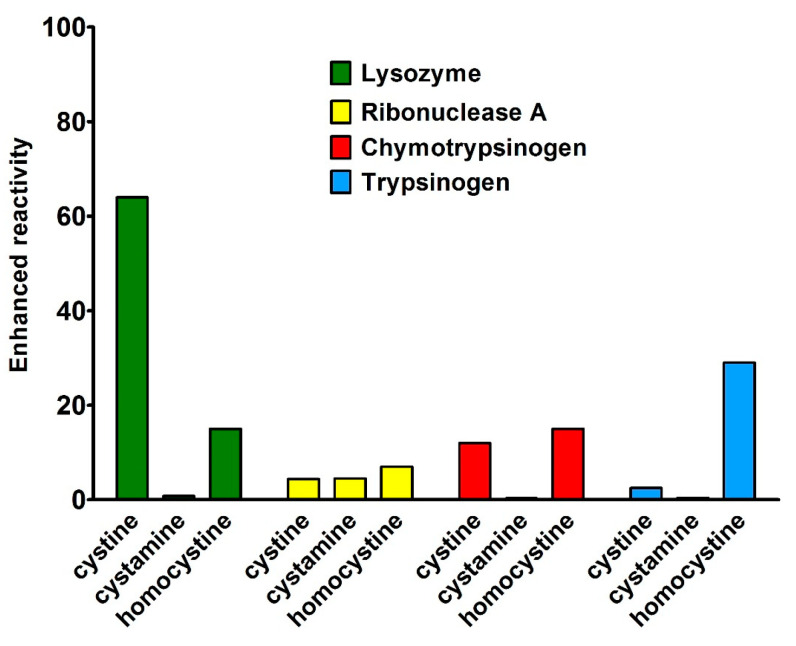
Reactivity of protein cysteines toward natural disulfides. The enhanced reactivity represents the second-order kinetic constants normalized to that of GSH. All proteins did not show any evident hyper-reactivity except the small enhanced reactivity found in Lysozyme toward cystine (65 times) which is small compared to the one of Cys94 toward GSSG (about 3000 times).

As a further important discovery is that scarce or no hyper-reactivity was observed toward other natural disulfides such as cystine, homocystine, and cystamine, confirming an almost exclusive specificity of interaction toward GSSG (Figure 3) [10,11,12,13].

Of particular interest is also the observation that similar hyper-reactivity is saved during a divergent evolution, as observed for chymotrypsinogen and trypsinogen, both coming from a common ancestral peptidase. This preservation during evolution was again a relevant clue for the implication of glutathione in the folding process.

**Figure 4:**
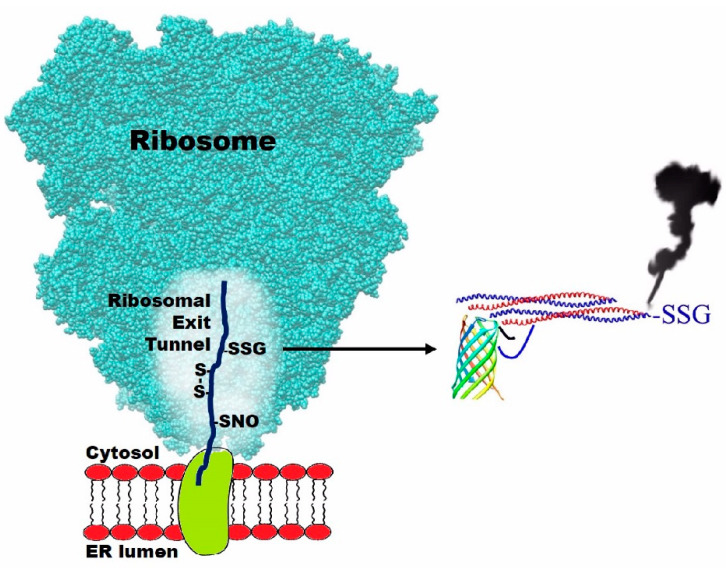
Visualization of ribosomal 50S subunit at the interface of endoplasmic reticulum with a nascent polypeptide chain. Modified cysteines of the bovine γB-crystallin found in the ribosomal exit tunnel during its nascent phase, as demonstrated in Ref. [15]. On the right, an “*imaginary joke structure*” of the glutathionylated protein, which represents the “smoking gun” for glutathione in the early scenario of the oxidative folding (the β-barrel structure represents the revolver grip, while the coiled coil is the revolver barrel).

These results demonstrate that the reduced molten globule conformations of all these proteins display a sophisticated propensity to interact with GSSG, a property typically unknown to the biochemist community. While this supports an early participation of glutathione in the folding pathway, it cannot be considered as final proof of it. A recent study, based on earlier studies [14] could, however, represent a decisive turn of this investigation [15]. It was in fact demonstrated that a nascent protein, the bovine γB-crystallin, could interact with glutathione in the ribosomal exit tunnel. Such protein, in fact, displays one of its seven cysteines (Cys18) either as a mixed disulfide with GSH or nitrosylated (Figure 4).

**Figure 5:**
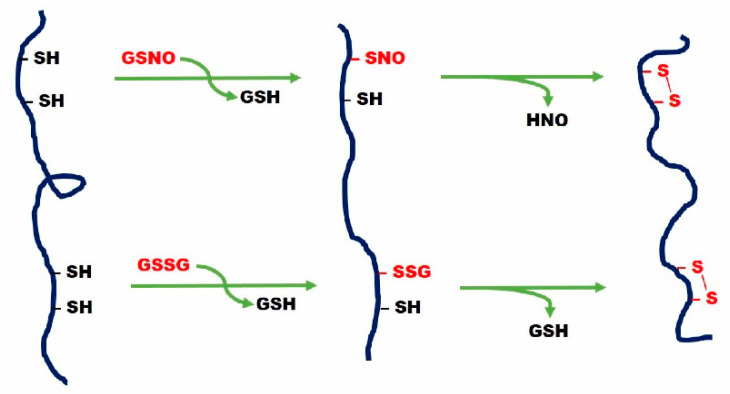
Schematization of modified cysteines on a polypeptide. The upper side represents the nitrosylation due to GSNO. The lower side represents the glutathionylation due to GSSG.

More surprisingly, detectable amounts of other cysteines have already been found in the form of disulfides (Cys15-Cys32; Cys22-Cys32; Cys32-Cys41; Cys15-Cys32) [15]. This finding provides strong evidence for the involvement of glutathione in the oxidative folding scenario. Apart from its presence as a mixed disulfide with Cys18, all the early protein disulfides found in this compartment are reasonably formed after a first glutathionylation or nitrosylation step caused by GSSG or S-nitrosoglutathione (GSNO) as represented in Figure 5.

In all these oxidative events no involvement can be evoked for PDI or Ero1: both these enzymes have not been found inside the tunnel and, more importantly, they cannot enter in this narrow ribosomal compartment having much more steric hindrance (diameter 50–60 Å for PDI and 40–60 Å for Ero1 as calculated from crystal structures) [16,17] compared to the one of the exit tunnel (diameter 10–20 Å) [15]. There is no reasonable objection that a similar phenomenon may occur in the nascent phase for many other disulfide containing proteins and this will be verified in the future.

A further interesting observation: the ribosomal synthesis of all proteins proceeds at about 20 amino acids/s and the synthesis of full-length γB-crystallin, made up of 174 amino acids, requires around 9 s. However, the tunnel contains only 34 residues [15] so the permanence of Cys18 as well as of the other cysteines in this compartment cannot exceed 1.5–2 s. Assuming that the Cys18 modification occurs only during its path through the ribosomal exit tunnel, we can consider 20 s to be a reasonable t_1/2_ for the nitrosylation and glutathionylation events. This value is easily estimated taking into account that 20% of Cys18 is found as a modified residue by NMR spectroscopy [15]. This putative t_1/2_ can be compared to the one resulting from the known kinetic constants for the reaction of GSSG and GSNO with a free cysteine (i.e., 0.7 M^−1^ s^−1^ [10] and 60 M^−1^ s^−1^ [18], respectively) and from the intracellular levels of these two compounds (0.4 mM for GSSG in the endoplasmic reticulum [5] and micromolar level for GSNO [19]). From these values, we can estimate much slower kinetics for both reactions (t_1/2_ ≈ 1–2 h). These data suggest a strong hyper-reactivity of Cys18 and other cysteines whose cause remains a fascinating enigma to be solved in the future. This property resembles the recently discovered hyper-reactivity toward GSSG of specific cysteines in the molten globular structures of albumin, lysozyme, ribonuclease trypsinogen, and chymotrypsinogen [8,9,10,11,12] but its origin is likely different. In fact, in the exit tunnel no globular structure of the protein can exist, thus no active-site-like cavity may be able to bind GSSG as it occurs in the molten globules of the above cited proteins. We can speculate that the internal membrane of the tunnel behaves like a proper surface able to catalyze the interaction of a few cysteines with GSSG and GSNO.

## 2. Conclusions

In conclusion, after twenty years from the first judgment, the criminal trial can be reopened to assess possible responsibility of glutathione at least in the early phase of the oxidative folding of several proteins. This does not exonerate PDI and Ero1 from any complicity in this scenario, but their involvement could be confined in a second phase after an initial very fast glutathionylation or nitrosylation step of a single or a few hyper-reactive cysteines triggered by GSSG or GSNO inside the ribosomal exit tunnel or in the endoplasmic reticulum as soon as the molten globule is formed.

## Figures and Tables

**Table 1:** Values of *K*_D_ for Protein-GSSG complex.

Proteins	*K*_D_ (mM)
Lysozyme ^a^	0.3 ± 0.1
Ribonuclease ^a^	0.12 ± 0.05
Chymotrypsinogen ^b^	1.5 ± 0.1
Trypsinogen ^b^	0.4 ± 0.1

^a^ Values obtained at pH 7.4 from Refs. [10,11]; ^b^ values obtained at pH 5.0 from Refs. [12,13].
